# Supplementary material for: Identification of photoperiod-induced specific miRNAs in the adrenal glands of Sunite sheep (Ovis aries)
Source: Front Vet Sci. 2022 Jul 22;9:888207. doi: 10.3389/fvets.2022.888207 (PMC9354845; doi:10.3389/fvets.2022.888207)
Supplement: Supplementary file 1 [file Data_Sheet_1.ZIP › Supplementary file/miRDeep2 file.zip!/miRDeep2 file/Novel_Structure/LP42A1/Novel_237.pdf]

Provisional ID	:	NC_019484.2_34861
Score total	:	2.7
Score for star read(s)	:	-1.3
Score for read counts	:	0
Score for mfe	:	2.4
Score for randfold	:	1.6
Score for cons. seed	:	
Total read count	:	103
Mature read count	:	103
Loop read count	:	0
Star read count	:	0

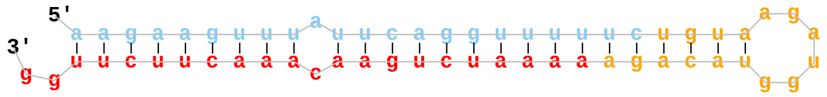

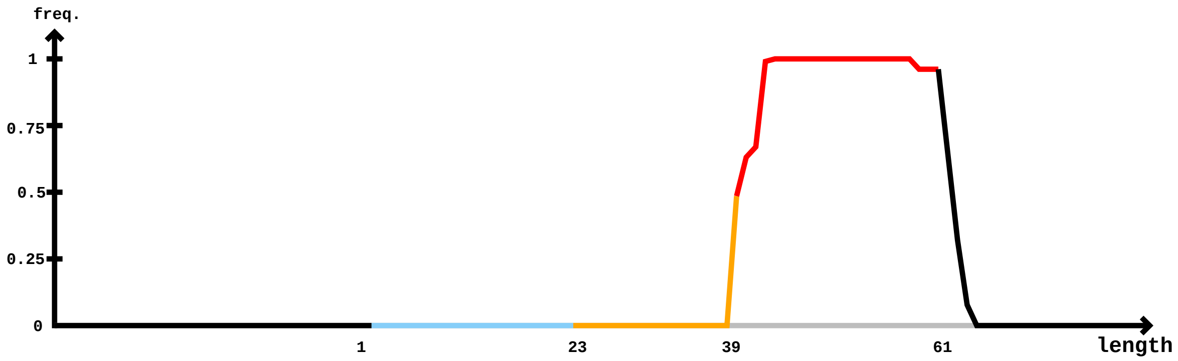

Star

Mature

```
5'- uucaucuguaauccaauauacuggguuggccaagaaguuuauucagguuuuucuguaagaugguacagaaaaaucugaacaaacuucuuggccaacccaauacuuaauucua   -3'    exp
    .....................(((((((((((((((((((.((((((((((((((((......)))))))))))))))).))))))))))))))))))).............     reads   mm   sample
    .....................................................................aaaaucugaacaaacuuUu........................     4       1    seq
    .....................................................................aaaaucugaacaaacuuUuugg.....................     45      1    seq
    .....................................................................aaaaucugaacaaacuuUuuggc....................     1       1    seq
    ......................................................................aaaucugaacaaacuuUuugg.....................     14      1    seq
    ......................................................................aaaucugaacaaacuucuuggc....................     1       0    seq
    .......................................................................aaucugaacaaacuuUuuggc....................     4       1    seq
    ........................................................................aucugaacaaacuuUuuggcc...................     25      1    seq
    ........................................................................aucugaacaaacuuUuuggcca..................     8       1    seq
    .........................................................................ucugaacaaacuuUuuggc....................     1       1    seq
```
